# Training opportunities of artificial intelligence (AI) in radiology: a systematic review

**DOI:** 10.1007/s00330-020-07621-y

**Published:** 2021-02-15

**Authors:** Floor Schuur, Mohammad H. Rezazade Mehrizi, Erik Ranschaert

**Affiliations:** 1 Vrije Universiteit Amsterdam, Amsterdam, The Netherlands; 2 School of Business and Economics, KIN Center for Digital Innovation, Vrije Universiteit Amsterdam, De Boelelaan 1105, VU Main Building A-wing, 5th floor, 1081 HV Amsterdam, The Netherlands; 3 Department of Radiology, Elisabeth-Tweesteden Hospital (ETZ), Tilburg, The Netherlands; 4 Ghent University, Ghent, Belgium

**Keywords:** Artificial intelligence, AI, Radiologists, Training, Curriculum

## Abstract

**Objectives:**

The aim is to offer an overview of the existing training programs and critically examine them and suggest avenues for further development of AI training programs for radiologists.

**Methods:**

Deductive thematic analysis of 100 training programs offered in 2019 and 2020 (until June 30). We analyze the public data about the training programs based on their “contents,” “target audience,” “instructors and offering agents,” and “legitimization strategies.”

**Results:**

There are many AI training programs offered to radiologists, yet most of them (80%) are short, stand-alone sessions, which are not part of a longer-term learning trajectory. The training programs mainly (around 85%) focus on the basic concepts of AI and are offered in passive mode. Professional institutions and commercial companies are active in offering the programs (91%), though academic institutes are limitedly involved.

**Conclusions:**

There is a need to further develop systematic training programs that are pedagogically integrated into radiology curriculum. Future training programs need to further focus on learning how to work with AI at work and be further specialized and customized to the contexts of radiology work.

**Key Points:**

*• Most of AI training programs are short, stand-alone sessions, which focus on the basics of AI.*

*• The content of training programs focuses on medical and technical topics; managerial, legal, and ethical topics are marginally addressed.*

*• Professional institutions and commercial companies are active in offering AI training; academic institutes are limitedly involved.*

**Supplementary Information:**

The online version contains supplementary material available at 10.1007/s00330-020-07621-y.

## Introduction

“AI isn’t going to replace radiologists, but radiologists who use AI will replace radiologists who don’t”*.*^1^

The introduction of artificial intelligence (AI) to radiology has created a sense of urgency among radiologists to learn about this new technology and its applications. Some surveys show that young medical professionals have recognized the major transformation that can happen to radiology [1] and have been in search of training opportunities to prepare them for the future [2]. Recent surveys show that most radiologists think that they should actively participate in training programs about AI as soon as possible [3] and they want to learn how to integrate AI into their work [4]. A recent international survey among 1041 radiologists shows that the majority of radiologists consider the lack of knowledge as a major hurdle for effective use of AI at work, thus demanding for AI-related training to be included in the residency curriculum.^2^

Scholars have stressed the need for training programs that introduce AI and its applications to radiologists in a professionally effective language [5], to help them understand how AI works with medical data and translates them into medical insights [3]. In addition, the rapid technological changes require the development of new training programs and swift update of their content. Next to the basics of medical data and informatics, advanced training on computer science and machine learning should be included in radiology curriculum [5].

Although AI training is not systematically integrated into their curriculum [4, 5], there have recently been various initiatives to train radiologists on AI-related subjects. The European Society of Radiology provides an online curriculum of radiology training (ESR-ETC),^3^ which includes learning “the functioning and application of Artificial Intelligence tools” as part of the broader “knowledge on different aspects of computer science and information technology in the field of medical imaging” in order to gain knowledge on “different technical options to implement AI and deep learning applications in the radiology workflow.” The European Society of Medical Imaging Informatics (EuSoMII) has supported the promotion and adoption of this curriculum for the integration of both technical and ethical aspects of deep learning into the radiology curriculum. EuSoMII has suggested different levels of knowledge and skills, which are now part of the ESR-ETC, with official credits to gain. However, the ESR-ETC is mainly a guideline and the actual training programs that need to materialize this guideline are not yet systematically incorporated in radiology curriculum in all member states.

Another notable initiative is the National Imaging Informatics Course (NIIC),^4^ co-organized by the Society for Imaging Informatics in Medicine (SIIM) and Radiological Society of North America (RSNA), which includes using algorithms in medical imaging and the foundations of deep learning. This program is offered to radiology residents, but is also open to PACS managers and other professionals in the medical imaging domain. In addition, American College of Radiology has been recently active in offering training programs on the foundations of AI and its potentials in radiology practice.

Besides, there are many training programs offered by a wide range of institutions. These programs are often occasional, short, and not integrated into the learning trajectory of radiologists. The fact that training radiologists on AI is quite emerging, there is still a major gap between the offerings of the training programs and what radiologists need to learn [4]. Radiologists and the training advisors are facing many scattered and occasional training materials, each have different focuses. They need to search for relevant training outside their official curriculum [3] and assemble their own learning elements often unsystematically. This runs the risk of being lost in heterogeneous, fragmented programs and missing a pedagogical framework and effective learning trajectory to offer the knowledge and skills that suit various groups of radiologists. So much so that some critics have questioned the common trend that urges all radiologists to learn data science, without critically considering their professional needs. Comparing AI with MRI, they suggest that “the new generation of radiologists should not necessarily become computer experts, but they should have a basic knowledge of the underlying technique” [6].

In this situation, we need a critical, systematic review of the AI training programs that are offered to radiologists. We need to systematically analyze their content, map out the various topics that are covered, and identify the missing ones. We also need to examine how these programs target different groups of radiologists to see how far they are specialized for their needs and their specific contexts. In addition, it is important to have an overview of the active agents and instructors in offering the training programs to examine how various institutional and professional institutions (e.g., medical, technological, and social) are involved. Finally, we should examine the ways in which training AI is promoted and legitimized as an important part of professional development for radiologists, especially when AI is not yet an official part of their training.

We conducted a systematic review of 100 AI training programs that are offered for radiologists in 2019 and 2020. Through the content analysis of these programs, we offer a systematic overview by examining (1) the topics that they cover and how actively they engage radiologists in learning about the application of AI in their work, (2) how specialized these programs are for various radiologists groups, (3) who are the active institutions in offering them, and (4) what are the ways in which the programs are legitimized as an important learning investment for radiologists. This analysis offers radiologists a systematic overview of the training opportunities that are available and enable them to critically select the relevant ones that suit their own needs. In addition, it helps managers and educational authorities see the available training programs that they can adopt and reflect on the further training programs that they can contribute to their development.

## Methods

We conducted a qualitative content analysis of the AI training programs that are offered to radiologists [7]. The research followed a process of search and selection, coding, and analysis.

### Search and selection

In this study, we use “training program” as an umbrella term for multiple formats in which a training is organized. It includes on-site and online courses, both educational seminars and webinars, and workshops or lectures. For being included, a program has to (1) be about artificial intelligence or other related topics such as deep learning, machine learning, and learning algorithms; (2) target radiologists as the audience; (3) have specific learning objectives; (4) specify the time and ways of delivery; and (5) have specific instructors. We therefore excluded the presentations, talks, videos, and other materials that did not have one of these criteria.

We searched for online information about the training programs, basically via Google, LinkedIn, and PubMed platforms, using a series of keywords (see Table 1). We also searched on the website of various radiological associations and organizations (see Table A6 in Appendix 1). We checked our initial list of training programs with four experts, who are active in offering AI training in radiology, which helped us enrich the list and find new programs. We initially found 130 potentially relevant programs. We excluded the programs about which limited information was available. Eventually, we collected a sample of 100 training programs, offered in 2019 and 2020 (until end of June), which appeared to cover a wide range of programs in terms of content and offering agents.

**Table 1:** Overview of the search platforms and keywords

Source	Explanatory notes	No. of training programs
LinkedIn	This platform provided me with lots of experts in the field of both radiology and AI who regularly posted initiatives for training programs.	52
Google	The first five result pages of Google were explored to look for training programs using the search terms described in the paragraph below this table.	38
PubMed	PubMed is a publicly accessible archive containing biomedical and science-related topics. The first five result pages of PubMed were explored to look for training programs using the search terms described in the paragraph below this table.	10

Search terms used within these sources: professional training program, dedicated training programs, educational programs, continuing education, complementary education, graduate medical education, hands-on training, study materials, followed by for radiologists, in the radiology domain, in medical imaging, in diagnostic imaging, for the new generation of radiologist, and finished with on artificial intelligence AI, machine learning ML, or deep learning DL

### Data collection and coding

We collected qualitative data about each training program by examining their general aspects (e.g., date, duration, and price), content, covered topics, target audience, instructors, offering agents, and ways of legitimizing (see Table 2). The qualitative data on each of these dimensions was archived in an excel file (see Appendix 2) for further analysis.

**Table 2:** The coding scheme

Dimension	Description	Contribution to empirical findings	Sub-categories
Date	The year in which the training program was held and the start date of the training program	To provide information about the date to include in the general overview of the training programs	Distinguishing between periods: - 2019—1st half - 2019—2nd half - 2020—1st half
Delivery mode	How the training program is offered to the trainees	The availability of the training in different places and times	- Offline - Online - Hybrid
Duration	The number of minutes, hours or days that a training program lasts	To provide information about the duration to include in the general overview of the training programs	- Shorter than 1 h - 1–3 h - 3–6 h - 1 day - 2 days - Longer than 2 days
Price	The amount of money a training program costs for participation	To provide information about the price to include in the general overview of the training programs	- Free - 0–100 EUR - 51–100 EUR - 101–200 EUR - 201+ EUR - Not specified
Content type	The type of content that is provided within the training program, more particularly: active or passive content	To distinguish between different kind of information that is provided in programs; theoretical, application, or hands-on content	- Passive-theoretical content - Passive-applied content - Active-hands-on training
Topic	The subjects on which the programs focus	To classify the content of the programs based on the topics they address and give indications about the popularity of each topic	Related to - Medical - Technical - Ethical/legal - Managerial
Audience	The people for whom the training is intended	To identify the intended people for whom the program is designed and what topics were common for this audience	- Only radiologists - Radiologists and other medical professionals - Radiologists and non-medical professionals - Radiologists and medical professionals and non-medical professionals
Instructors	The speakers that teach the content of the programs	To find out what instructors teach on programs, see the link with the topics discussed and see if the instructors can be linked to an indication of legitimacy	Different instructors’ backgrounds: - Medical - Technical - Other
Offering Agent	The institution that offers the training program	To examine how different institutions such as professional, academic, and commercial are active in offering the training programs	- Professional institutions - Academic institutions - Commercial companies - Other
Legitimization	How the programs legitimize their training programs	To distinguish between the different ways of achieving legitimacy for their training program	Legitimizing based on: - Content - Offering agent - Accreditation - Acknowledgement

We used deductive thematic analysis [8] to analyze the data, based on the codebook (Table 2). One coder coded the entire data and a second coder cross-checked the results and resolved the ambiguities. The results were organized in a database (Appendix 2), in which we explored patterns based on cross-tabulation and comparisons between the training programs [9]. We interacted with three experts in the domain of AI and radiology to validate the relevance and richness of the extracted patterns.

## Findings

### Overview of the AI trainings

**Fig. 1:**
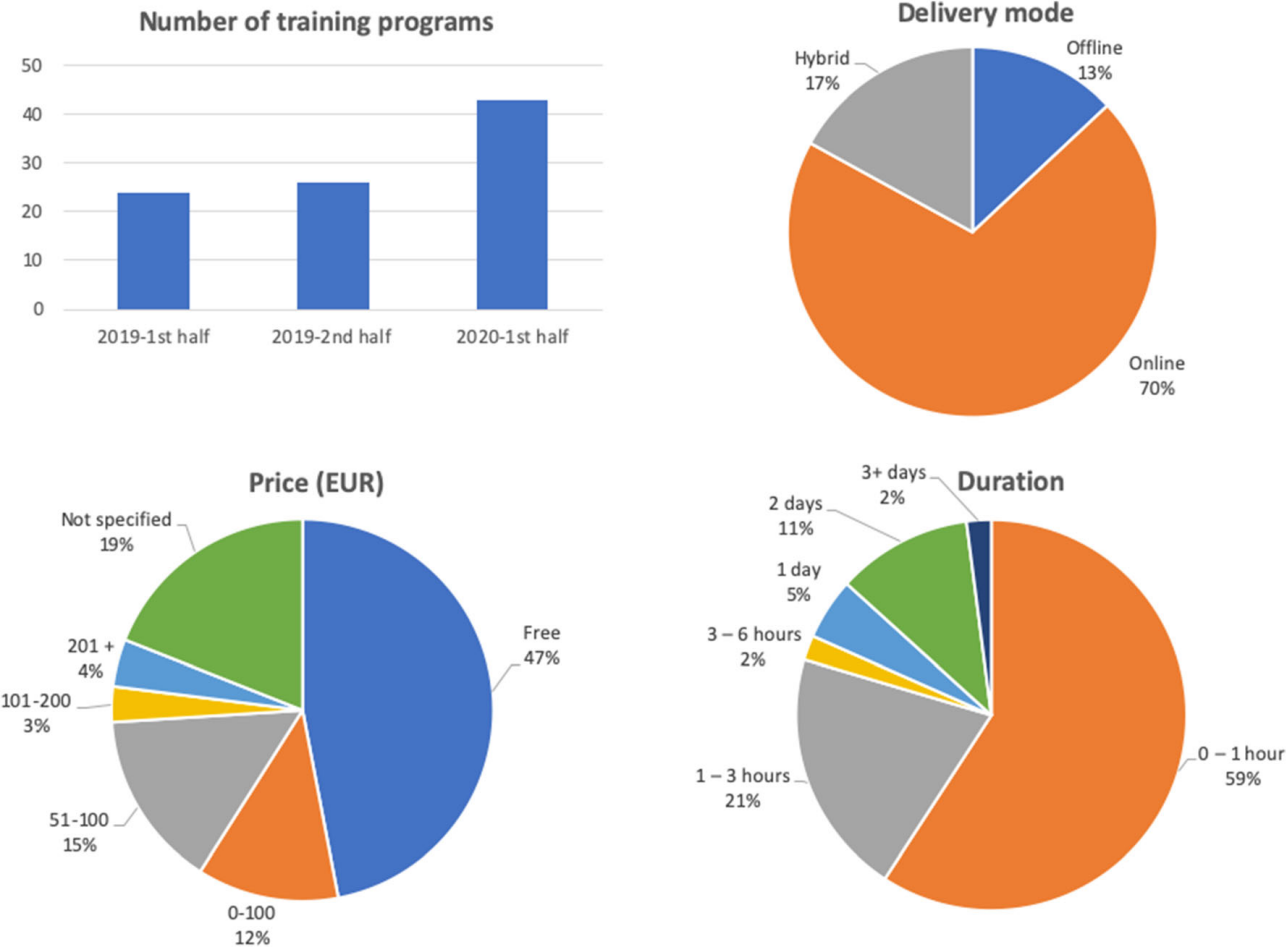
The overview of the training programs

The number of AI trainings for radiologists has increased in the last 1.5 years (Fig. 1). Most of the training programs are offered purely online (70%), and only a small fraction of them are offline (13%). Half of these programs are offered for free; for the rest, the average price per program is less than 100 Euro. Around 60% of the programs are shorter than 1 h and only 20% of them take longer than 3 h. Overall, most of the programs are short, stand-alone sessions, which are not part of any larger curriculum (see Tables A1 and A2 in Appendix 1).

### The content of training programs

**Fig. 2:**
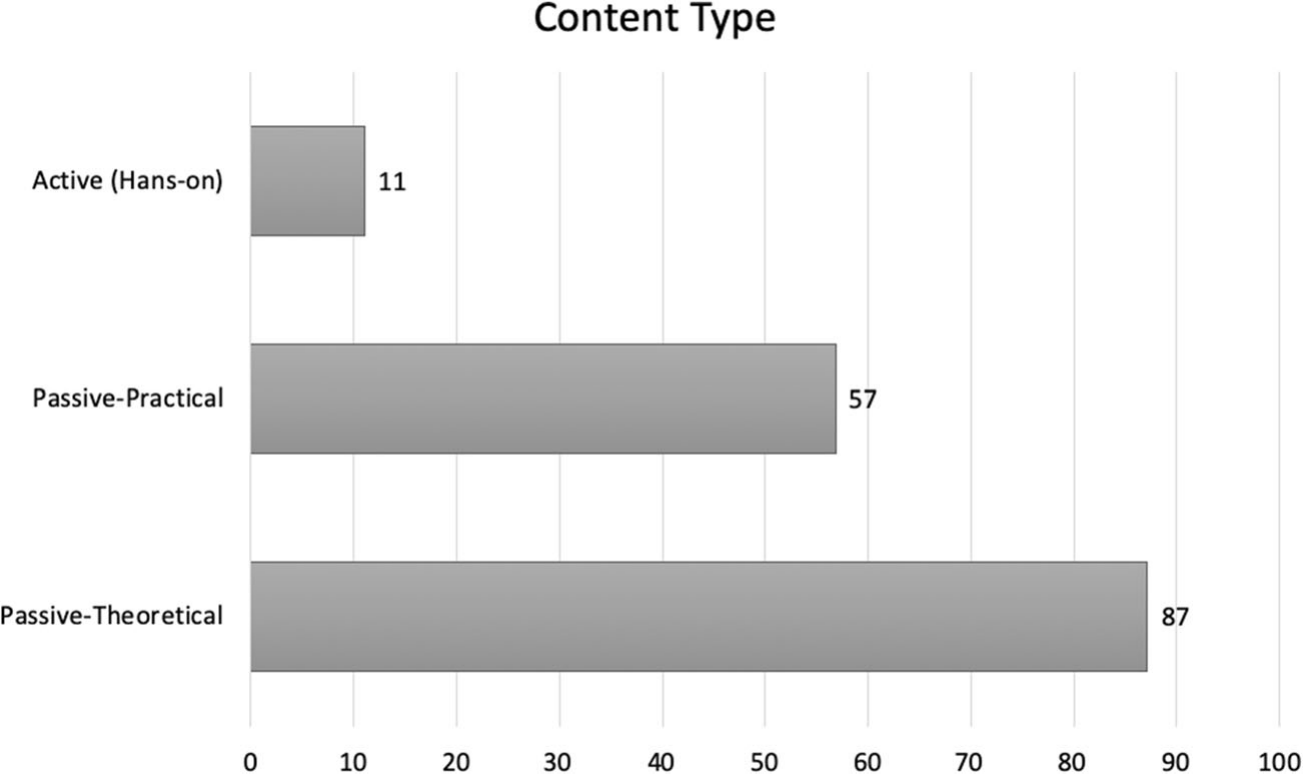
Prevalence of the various content types in the training programs

The analysis of their content (Fig. 2) shows that a majority of the training programs focus on passive offering of the content about the conceptual aspects of AI such as the basics of machine learning and its potentials for medical practice in general (not necessarily related to radiology). Around half of the programs go further and discuss some potential applications of AI in radiology, e.g., by showing how artificial intelligence can be applied to diagnostic imaging, and how AI can be applied to a particular modality (CT, MRI) or giving examples of AI applications in clinical radiology. However, only a small fraction of the programs practically engages the learners in hands-on practices of working with some tools. There, the focus is on practicing the basics of writing code (e.g., programming in Python) or training an algorithm based on some image data. Few programs focus on learning how to perform the actual medical work with real AI applications (see Table A3 in Appendix 1).

We clustered the topics that these programs cover into (1) medical (how AI can support medical diagnosis), (2) technical (how AI can be developed, tested, and configured), (3) legal/ethical (what are the legal and ethical considerations of implementing and using AI), and (4) managerial (e.g., integration of AI in the radiological workflow, how to best deploy AI and the inherent challenges) aspects (see Table A4 and Fig. A1 in Appendix 1). Overall, the focus of many programs is on a combination of medical and technical topics, sometimes combined with peripheral discussions about the legal/ethical and/or managerial aspects of implementing AI. Although managerial topics are covered in around half of the programs, they are often side discussions.

### Target audience

**Fig. 3:**
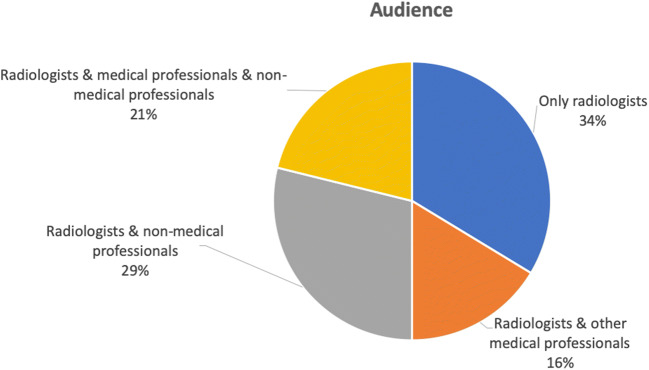
Target audience of the training programs

Although we selected the training programs that are offered to radiologists, most of them (76%) target other medical professionals (e.g., other specialization, GPs, medical researchers) and even non-medical professionals (e.g., data scientists, IT specialists, managers) as well (Fig. 3). In fact, most of the programs are quite generic (e.g., on the basics of AI), thus are promoted as relevant for a wider set of audience than merely radiologists. Only in 7% of the programs radiographers are explicitly included as the audience. When training programs merely focus on radiologists, their content becomes more connected to the medical cases and the integration of AI into the daily workflow.

### Instructors and offering agents

**Fig. 4:**
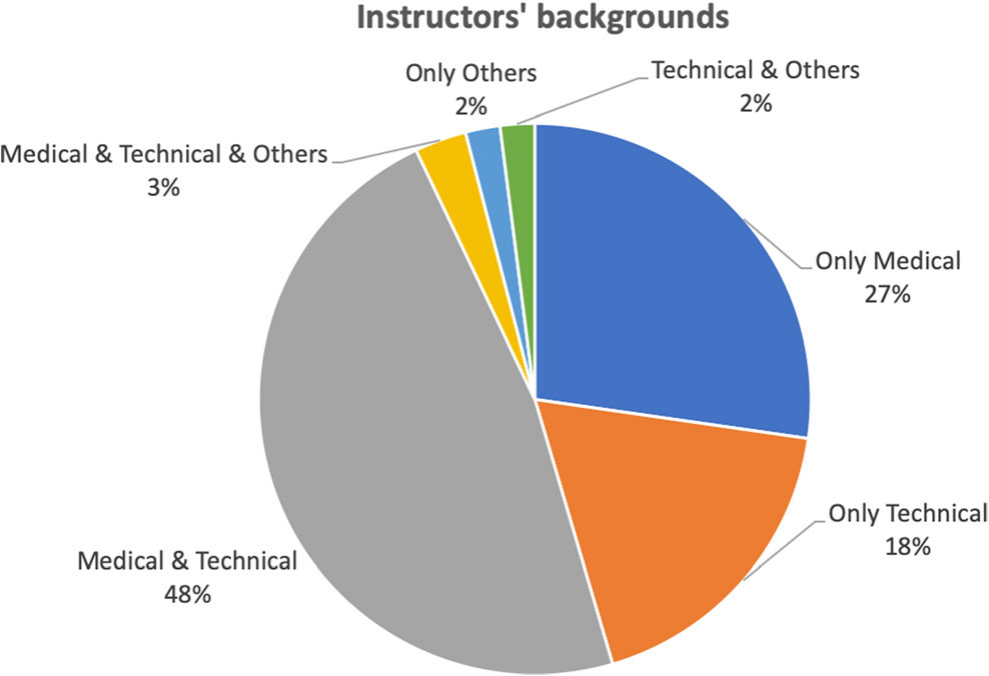
Instructors’ backgrounds

The instructors have very diverse backgrounds such as “professors of radiology,” “founders of AI companies,” and “researchers” from R&D departments. Half of the programs are jointly offered by the instructors with medical and technical backgrounds (Fig. 4). Only one-third of the programs are offered by radiologists and very few instructors come from the social and managerial domains (see Table A5 in Appendix 1 for an overview of the instructors’ backgrounds). Sometimes, people with medical background offer technical content (e.g., basics of machine learning).

**Fig. 5:**
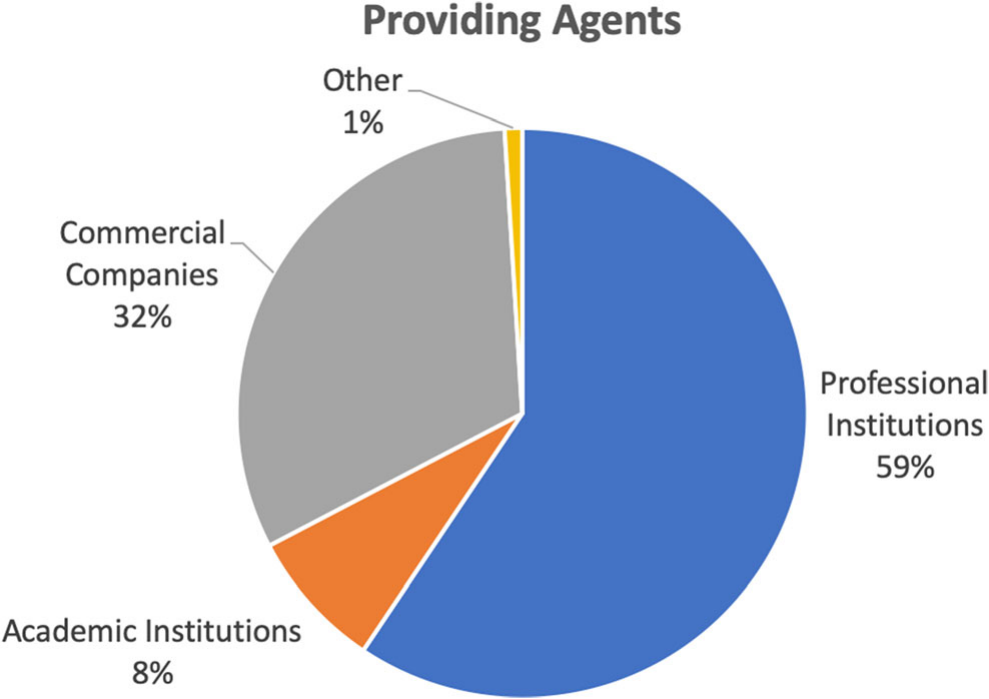
Providing agents

The providing agents (Fig. 5 and Table A6 in Appendix 1) are primarily professional institutions such as radiology associations and societies (59%). Commercial companies such as AI developers and commercial training agents are also active in offering AI. Academic institutes are marginally involved (only 8%).

### Legitimization strategies

**Fig. 6:**
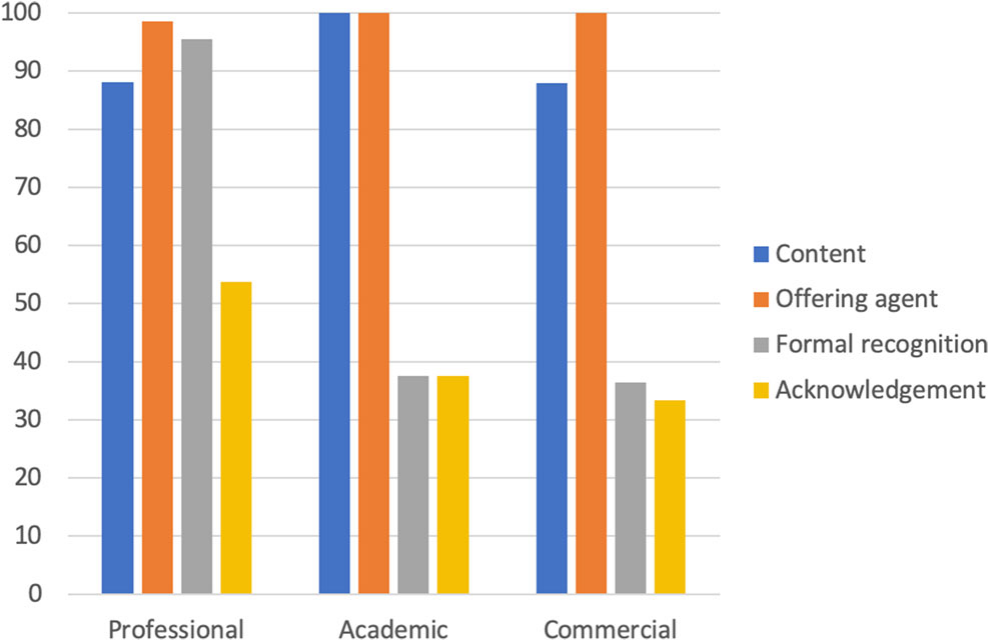
Legitimization strategies used by different providing agents

Since AI is not officially part of the radiology curriculum yet, the providing agents need to use various strategies to legitimize their programs as relevant and valuable for radiologists (see Table A7 in Appendix 1). Our analysis shows that most of the training programs use multiple strategies to legitimize their programs, including (1) showing the importance of the content (90%), (2) appealing to the status of the providing agents and instructors (99%), (3) offering accreditations and certificates for participants (73%),^5^ and (4) showcasing the positive feedback and acknowledgements of the prior participants (47%). Half of the formal recognitions that these programs offer are medically recognized accreditations such as CME, approved by EACME or AACME. As Fig. 6 shows, professional institutions are using the first three strategies to a high extent, whereas academic and commercial institutions mainly focus on legitimizing their programs based on the content and the status of the providing agents.

## Discussions

As AI becomes increasingly important, radiologists need to actively engage in learning about it and acquire the skills that enable them to effectively use AI.

### From scattered, fragmented trainings to systematic curriculum

Our analysis shows that most AI training programs offered to radiologists are primarily short, stand-alone, and fragmented.^6^ Although they are useful for creating awareness and familiarizing radiologists with basic concepts, they still need to be complemented with programs that are coherent and pedagogically integrated into the general radiology curriculum. As we are in the stage of reframing the radiology profession, we need to develop training programs that are based on scientific research and offer an effective learning trajectory for radiologists who want to invest in long-term development of their career. Some initiatives such as the ESR-ETC can serve as guiding frameworks for designing the programs that complement each other. This requires a more active involvement of academic institutes in designing and offering the training programs that are based on scientific research and aligned with pedagogical principles.^7^ When these training opportunities are offered systematically as part of the national radiology curricula, they could be incorporated into professional examinations such as European Diploma in Radiology (EDiR). Here, some regional and international institutions such as ESR can play a strong role by offering relevant courses needed for the EDiR examination.

### From awareness to learning how to work with AI applications in clinical settings

Our findings show that the majority of the programs focus on passive delivery of content on the basics of AI and its potential impacts on radiology work. As AI gradually finds its way in the daily practice of radiologists, we need to engage learners in practical exercises with real AI applications and learning how to use them effectively and critically at their work. Training programs need to focus more on real use-cases, and (close to) real work settings and applications. Learning about the basics of machine learning, though might offer some basic knowledge, is not enough for radiologists who will be “working with,” rather than “developing,” the AI applications. Learning how to critically use and effectively integrate AI applications into the working routines requires developing new mental and practical skills. Developing these skills requires the involvement of not only medical and technical instructors, but also experts from organizational, legal, ethical, and psychological domains. Above all, younger radiologists need to be supported by programs that enable them to strategically design and develop their professional career for future.

### From generic to specialized and customized training

Finally, our study shows that most of the current training is generic in terms of their content and target audience. Although these generic programs are effective for creating some basic familiarity, they are limited to develop knowledge and skills that are specific to the context of radiology work. Further programs can be more specialized on the specific use-cases that radiologists face in using AI and consider the special needs of radiologists (e.g., when residents need to check their examinations with a senior radiologist). Furthermore, we need to develop training programs that are customized and localized to the specific clinical practices and organizational settings. Especially in Europe, the working conditions, legal frameworks, and workflow configurations vary from one country to another. All these differences require customized training programs that go beyond the generic ideas and consider the specific organizational settings (e.g., technological infrastructure), legal frameworks (e.g., privacy regulations), and cultural aspects (e.g., patients’ and referring physicians’ expectations of and trusts in AI applications). Table 3 offers a list of suggestions for developing AI training.

**Table 3:** Suggestions for developing and selecting appropriate AI training programs for radiologists

• Integrating the basics of machine learning and its applications into radiology curriculum • Covering a pedagogical path through which learners accumulate their knowledge • Combining passive (offering content) and active (hands-on) elements • Paying balanced attention to the basics of machine learning and clinical application in radiology • Paying attention to the practical implementation in clinical settings, including the integration of AI with the existing systems and infrastructure • Covering the managerial, contractual, and financial aspects (e.g., how to define feasible business cases, how to estimate ROI and decide on the reimbursement model, and how to define value propositions) • Considering ethical, legal, and organizational aspects of working with AI (e.g., privacy and security considerations in handling medical data) • Being supported by academic institutes and scientific communities • Being customized (e.g., for sub-specializations) and localized into the specific working conditions and legal environments • Integrating the practical and supporting components to deepen the learning (e.g., career coaching, mentoring, …)	

### Limitations and future research

Although we examined a diverse sample of training programs, our focus on LinkedIn (instead of Twitter) may have biased our sample towards Europe [10]. Future studies can also examine the other offerings such as podcasts and knowledge clips, which, although are not officially framed as training programs, are often used by radiologists to learn about AI [11]. In addition, since many training programs are still emerging, future studies need to continuously examine new trends and update our findings. Next to radiologists, other professional groups such as radiographers, clinical physicists, and technical physicians are learning how to use AI for supporting various tasks in the acquisition of the images and pre-processing them. Knowing how they learn to work with AI and what skills they can develop is important to understand how AI can be effectively used in radiology.

## Supplementary Information

ESM 1 Detailed overview of the training programs (DOCX 185 kb)

ESM 2 Database of the training programs is offered as an excel file (as a supplementary material) (XLSX 98 kb)

## Abbreviations

ACCME: Accreditation Council for Continuing Medical Education
AI: Artificial intelligence
CME: Continuing Medical Education
CT: Computed tomography
EACME: The European Accreditation Council for Continuing Medical Education
EDiR: European Diploma in Radiology
ESR: European Society of Radiology
ESR-ETC: ESR European Training Curriculum
EUSOMII: The European Society of Medical Imaging Informatics
MRI: Magnetic resonance imaging
NIIC: National Imaging Informatics Course
PACS: Picture Archiving and Communication System
R&D: Research and development
RSNA: Radiological Society of North America
SIIM: Society for Imaging Informatics in Medicine

## Footnotes

1 Curtis Langlotz, posted on Twitter on Feb 8, 2017

2 The study is not yet published: Huisman M, Ranschaert E, Parker W, et al An international survey on AI in radiology in 1041 radiologists and radiology residents part 2: expectations, hurdles to implementation and education

3 https://www.myesr.org/education/training-curricula

4 https://siim.org/page/niic

5 Formal accreditation is an important motivation for radiologists who need to collect a series of credits/points to be able to renew their professional eligibility.

6 See NIIC-RAD program as an exception.

7 See the online training on “Fundamentals of Machine Learning for Healthcare,” from Stanford Uni, as an example of training programs developed with regard to radiologists’ needs.
