# Supplementary material for: Training opportunities of artificial intelligence (AI) in radiology: a systematic review
Source: Eur Radiol. 2021 Feb 15;31(8):6021–9. doi: 10.1007/s00330-020-07621-y (PMC8270863; doi:10.1007/s00330-020-07621-y)
Supplement: Supplementary file 1 — Detailed overview of the training programs (DOCX 185 kb) [file 330_2020_7621_MOESM1_ESM.docx]

Figure A1. Share of various topics covered by the training programs


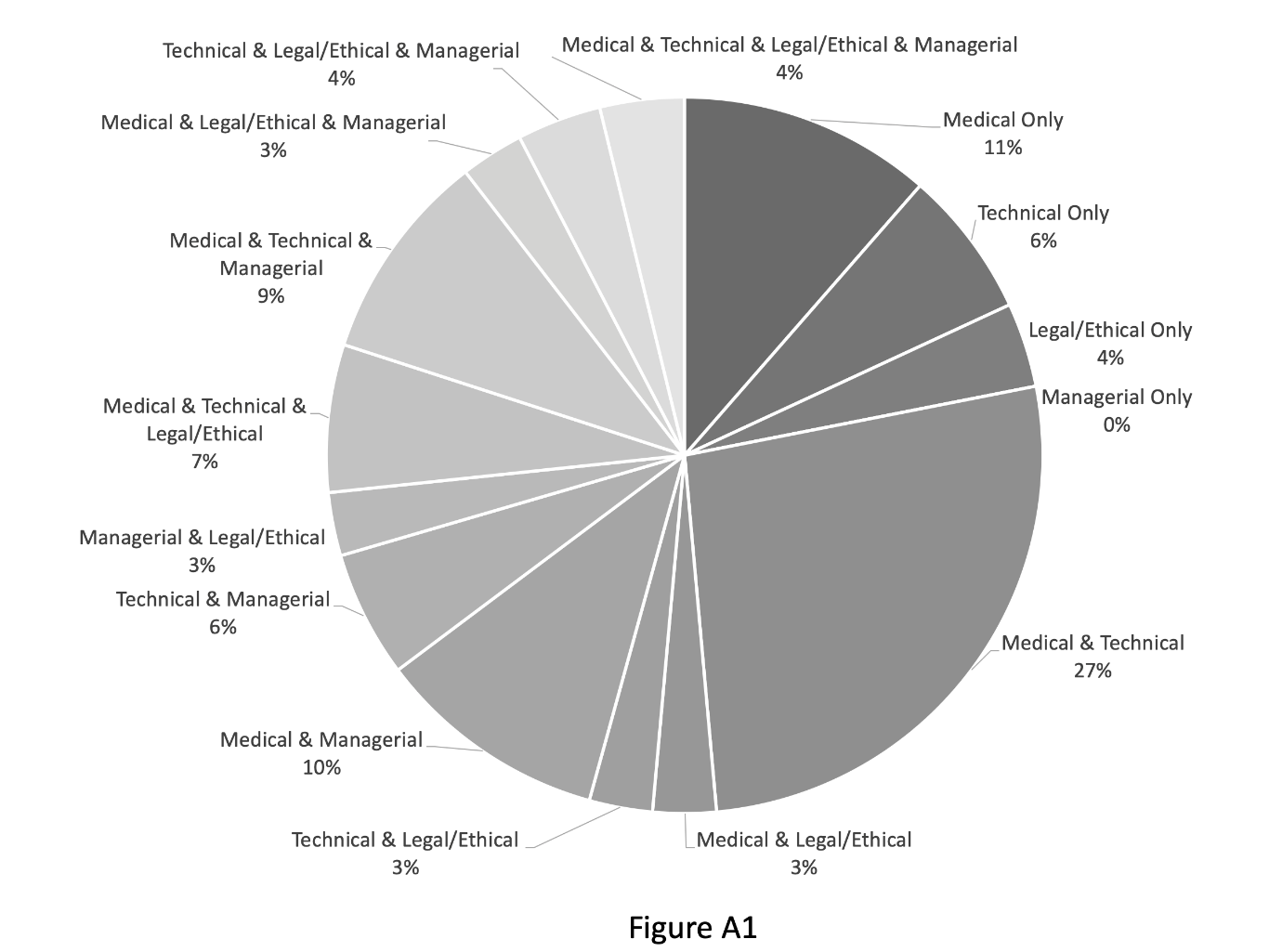


Table A1. Price of training programs

| **Price** | | |
| --- | --- | --- |
| Free | 47 programs | Explanatory notes: some training programs take place within a larger event such as a conference. These training programs’ prices are assessed based on the price of the event, since that amount is required to be paid to access the training program. In that context, some programs need to be bought in combination which provides access to a package of training programs rather than just one. Here, the total package price has been considered since that amount is required to access the program, even if it’s only one.    Some affiliations do not mention anything about their prices. Although no hasty conclusion can be drawn, this may indicate that providing agents deliberately hide their prices because of underlying motives. |
| 0-50EUR | 12 programs |  |
| 50-100EUR | 15 programs |  |
| 100-200EUR | 3 programs |  |
| 200-400EUR | 2 programs |  |
| 400 + EUR | 2 programs |  |
| Not specified | 6 programs |  |

Table A2. Duration of training programs

| **Duration** | | |
| --- | --- | --- |
| 0 – 1 hour | 58 programs | Explanatory notes: the training programs with long duration (1 day or longer) are courses specifically designed for AI training on radiologists. Based on their specified and qualified content, they are considered as appropriate training programs. Some of these programs could not be broken down into smaller ones. This may explain the differences in duration. One day equals approximately eight hours. |
| 1 – 3 hours | 20 programs |  |
| 3 – 6 hours | 2 programs |  |
| 1 day | 5 programs |  |
| 2 days | 11 programs |  |
| 20 hours | 1 program |  |
| 7 days | 1 program |  |

Table A3. Different content types

| **Content** | | **Description** | **Illustrative finding** |
| --- | --- | --- | --- |
| Passive | Theoretical | Discussing theoretical concepts of radiology, technologies or other topics that are not related to the application of AI | “*General concepts of machine and deep learning will be explored*” (ESR – Intelligence, Innovation, Imaging – The Perfect Vision of AI)  “*Tips on knowing the use cases, emphasizing the expected value of AI, and familiarize with the organization's operational state and business goals*” (SIIM – AI Implementation) |
|  | Application | Discussing actual applications of AI in radiology, explanation of these applications and modality-specific examples | “*How the basic technical principles of AI are applied to diagnostic imaging and clinical applications*” (ESR)  “*The application of AI explained in steps for image acquisition and image reconstruction, segmentation, identification of anatomical structures*” (EuSoMii – Machine Learning in Cardiovascular imaging) |
| Active | Hands-on | Practicing with knowledge and skills learned and apply it into clinical practice | “*Gain practical skills to improve patient care in your practice to advance your practice*” (RSNA – Radiology in the Age of AI)  “*The first module introduces data sets for a concrete AI project in Medical Imaging. It teaches participants on how to use tools to prepare imaging data for analysis with Artificial Intelligence.”* (Sitem Insel – Artificial Intelligence Project) |

Table A4. Overview of topics covered in the training programs

| **Topic** | **Description** | **Example** | **Included in:** |
| --- | --- | --- | --- |
| Medical | How AI is influencing the clinical radiology practice including specific modalities or radiology specializations | “*the clinical applications of a cutting-edge brain imaging AI in the evaluation of patients with dementia, multiple sclerosis and traumatic brain injury*” (AIMed – Transforming patient care through imaging AI) | 75 programs |
| Technical | Discussing the foundations and both the basic and advanced techniques of AI technology and related technologies | “*Includes a general tutorial on machine learning that aims to convey the importance of AI.”* (SCCME – Artificial Intelligence and Machine Learning in MSK Radiology) | 69 programs |
| Managerial | Elaboration of implementation and integration of AI in the radiology department and workflow | “*Gain unique insights into how organizations can overcome the challenges through a platform approach to AI implementation in imaging workflows that makes it seamless*.” (GE Healthcare – How to Seamlessly Integrate AI into your Imaging Workflows) | 40 programs |
| Legal/Ethical | Questions that are emerged through AI regarding legal, ethical and social issues | “*Explain hurdles and steps to regulatory clearance and define evaluations of AI and common issues including bias, brittleness, and fairness*” (ACR – Monitoring and Evaluating AI: Challenges and Practical Implications) | 29 programs |

Table A5. Backgrounds of the instructors

| **Radiologist** | Diagnostic Radiology  Diagnostic Imaging  Radiology  Neuroradiology  Radiology Informatics  Musculoskeletal Radiology  Breast Imaging  Cardiothoracic Radiology  Cardiovascular Imaging  Interventional Radiology  Emergency Radiology  Paediatric Radiology |
| --- | --- |
| **Other Medical Professionals** | Professor of Medicine  Researcher in Translational Neuroscience  Physician Scientist  Clinical Scientist  Medical Doctor |
| **Technical Background** | Computational Imaging  Biomedical Engineer  Biomedical/Medical/Diagnostic Image Analysis  Medical Image Analysis  Diagnostic Physics and Imaging Informatics  Medical/Imaging/Radiology/Clinical Informatics  Pathology Informatics  Physics in Medical Data  Medical Physicist  Biomathematics, Biostatistics and Human Genetics  Medicine and Tropical Diseases  Bioengineering, Of Genetics, Of Medicine    AI Expert  Computer Science  Data Scientist  Machine Learning Engineer  Statistical Methods  Computer Vision Engineer  Information Technology    Electrical Engineering  Human Centered Design & Engineering |
| **Other Backgrounds** | Medical Manager (E.G., Chief Medical Officer, Clinical Health Sciences)  Medical Entrepreneur  Medical Consultant (E.G., Consultant-Healthcare Strategies, Marketing Strategy and Product Development.)  Healthcare Policy and Research  History of Medicine |

Table A6. list of providing agents and the number of training programs

| **Providing Agents** | **Professional Institutions** | **Academic** | **Commercial** | **Other** |
| --- | --- | --- | --- | --- |
| AI in Healthcare |  | 2 |  |  |
| AIDoc |  |  | 5 |  |
| AIMed |  |  | 4 |  |
| American College of Radiology ACR | 7 |  |  |  |
| American Osteopathic College of Radiology AOCR | 1 |  |  |  |
| Applied Radiology Expert Forums |  |  | 1 |  |
| Aunt-MinnieEurope |  |  | 2 |  |
| British Institute of Radiology | 3 |  |  |  |
| Coursera |  |  | 1 |  |
| European School of Radiology ESOR | 1 |  |  |  |
| European Society of Medical Imaging Informatics (EuSoMII) | 8 |  |  |  |
| European Society of Radiology ESR | 15 |  |  |  |
| GE Healthcare |  |  | 2 |  |
| iCad |  |  | 2 |  |
| Indian Radiological & Imaging Association | 1 |  |  |  |
| M&I Partners |  |  | 1 |  |
| Noord West Ziekenhuisgroep |  |  |  | 1 |
| North West Radiology Network NWRN | 1 |  |  |  |
| Quantib |  |  | 2 |  |
| Radiological Society of North America RSNA | 5 |  |  |  |
| Radiology Business |  |  | 2 |  |
| Radsite |  |  | 1 |  |
| Sitem Insel |  |  | 6 |  |
| Skyl.ai |  |  | 2 |  |
| Smart Health |  |  | 1 |  |
| Society for Imaging Informatics in Medicine SIIM | 10 |  |  |  |
| Society of Nuclear Medicine and Molecular Imaging SNMMI | 6 |  |  |  |
| Stanford Center for Continuing Medical Education; SCCME |  | 2 |  |  |
| University Hospital Zürich UHZ |  | 1 |  |  |
| University of Toronto |  | 1 |  |  |
| UT Southwestern Radiology | 1 |  |  |  |
| VUB Artificial Intelligence Lab |  | 2 |  |  |
| **Total** | 59 | 8 | 32 | 1 |

Table A7 Legitimization strategies used for promoting the training programs

| **Strategies** | **Description** |
| --- | --- |
| Content | How do the providing agents show that the **content** of their training programs is important and relevant? |
| Offering Agent | How do the providing agents show that **they** are suitable to offer the training programs? |
| Formal Recognition | How do training programs show that they have been **formally recognized** (e.g., by receiving a certificate)? |
| Acknowledgement | How do the providing agents show that the **stakeholders (e.g., learners)** have been satisfied with the training programs? |

Table A8. List of the training programs (ordered based on the offering agents)

| **Training Program** | **Offering Agent** |
| --- | --- |
| Architecting AI: Rethinking Medical Imaging & Defining the Strategy | AI in Healthcare |
| Architecting AI: Why Machine Learning is Changing Medical Imaging | AI in Healthcare |
| Is AI ready to become standard of care? | AIDoc |
| Integrating AI into the Radiology Workflow - Dos and Dont's | AIDoc |
| Deep Learning for Medical Imaging | AIDoc |
| Showing the value of AI - Quality in the radiology workflow | AIDoc |
| Beyond the Hype; AI in Practice | AIDoc |
| Radiology: Pragmatic Solutions for Ever-Evolving Areas of Medicine | AIMed |
| Continuing the discussion for successful translation of AI into clinical radiology | AIMed |
| Transforming patient care through imaging AI | AIMed |
| Breaking through the bottlenecks: successful translation of AI into clinical radiology | AIMed |
| Monitoring and Evaluating AI: Challenges and Practical Implications | American College of Radiology |
| Current State of AI in Practice: Diverse Perspectives and Panel Discussion | American College of Radiology |
| How Enterprise Imaging Intersects Enterprise AI Development | American College of Radiology |
| Update on Regulatory and Reimbursement Challenges with AI | American College of Radiology |
| Data Access, Privacy and Security of AI | American College of Radiology |
| Optimizing the IT Supply Chain to Deploy AI in the Clinical Workflow | American College of Radiology |
| ACR AI-LAB: bringing AI to Routine Clinical Practice: Concepts and Demonstration | American College of Radiology |
| Leveraging Artificial Intelligence to Improve Radiologist Reporting Quality & Efficiency | American Osteopathic College of Radiology |
| Leveraging Artificial Intelligence in a COVID-19 Environment | Applied Radiology Expert Forums |
| Virtual Conference Radiology | Aunt Minnie |
| Making AI Meaningful: How AI Is Being Used To Transform Radiology Practices | Aunt Minnie |
| Artificial Intelligence in Radiology 2020 | British Institute of Radiology |
| Artificial Intelligence in Radiology 2019 - where are we now | British Institute of Radiology |
| AI in radiology: the main worries | British Institute of Radiology |
| AI for Medical Diagnosis | Coursera |
| Foundation Course on Artificial Intelligence in Radiology. | European School of Radiology |
| Data sharing and analytics platforms for medical imaging in the age of big data | European Society of Medical Imaging Informatics |
| Preparing medical imaging data for machine learning | European Society of Medical Imaging Informatics |
| Integration of AI in radiological workflow | European Society of Medical Imaging Informatics |
| From Idea to Product: AI Based Applications in Radiological Breast Imaging | European Society of Medical Imaging Informatics |
| Machine Learning in cardiovascular imaging | European Society of Medical Imaging Informatics |
| AI & Neuroimaging | European Society of Medical Imaging Informatics |
| AI & MSK Imaging | European Society of Medical Imaging Informatics |
| AI to the clinic | European Society of Medical Imaging Informatics |
| Radiology fighting COVID-19: How artificial intelligence can help | European Society of Radiology |
| Intelligence, Innovation, Imaging - The Perfect Vision of AI | European Society of Radiology |
| Artificial Intelligence: presented with new lecture formats | European Society of Radiology |
| Artificial intelligence (AI): driven by radiologists | European Society of Radiology |
| Artificial intelligence (AI): our future cannot be predicted, but we have to be | European Society of Radiology |
| Artificial intelligence in hybrid imaging | European Society of Radiology |
| 1: Basics of Machine Learning and Deep Learning for AI in Medicine | European Society of Radiology |
| 2: Machine Learning in medical imaging going forward | European Society of Radiology |
| 3: How to integrate AI technology in radiology today | European Society of Radiology |
| 4: The impact of AI technologies in patient care: advantages and limitations | European Society of Radiology |
| 5: How will the introduction of AI change the role of the…? | European Society of Radiology |
| 1: The one where we classify breast cancer with MRI | European Society of Radiology |
| 2: The one where we tackle fibrosis | European Society of Radiology |
| 3: The one where we make the connection | European Society of Radiology |
| 4: The one with whole body MRI | European Society of Radiology |
| The role of the CT & AI in the detection, diagnosis & follow-up of COVID-19 | GE Healthcare |
| How to Seamlessly Integrate AI Into Your Imaging Workflows | GE Healthcare |
| How Radiologists can Easily and Effortlessly Integrate Artificial Intelligence in Breast Screening During and After the COVID-19 | iCad |
| Assisting Radiologists: How can AI improve the efficiency of breast healthcare after COVID-19? | iCad |
| What do tech companies want from radiologists? | Indian Radiological & Imaging Association |
| AI in Radiology | M&I Partners |
| Leergang Data Science for Healthcare | Noord West Ziekenhuisgroep |
| What Keeps Radiology Administrators Up at Night? | NorthwestRadiologyNetwork |
| How AI can help with quantification in radiology | Quantib |
| How to successfully and responsibly introduce AI in neuro-radiology | Quantib |
| Intro to AI and Machine Learning: Why All the Buzz? | Radiological Society of North America |
| Current State and Future Perspectives of AI | Radiological Society of North America |
| AI: An Ally or an Enemy? A roundtable discussion | Radiological Society of North America |
| Radiology in the Age of AI | Radiological Society of North America |
| Demystifying Machine Learning and Artificial Intelligence for the Radiologist | Radiological Society of North America |
| Radiology Technology Innovations: AI and Beyond | Radiology Business |
| Artificial Intelligence Adoption in Imaging and Informatics, and Why Infrastructure Matters | Radiology Business |
| Artificial Intelligence in Medical Imaging: Hype, Reality and Future Applications | Radsite |
| Artificial Intelligence Project | Sitem Insel |
| Statistics and Programming | Sitem Insel |
| Fundamentals in AI | Sitem Insel |
| Applied AI in Medical Imaging | Sitem Insel |
| Applications of AI | Sitem Insel |
| Legal and Ethical Challenges of AI | Sitem Insel |
| AI in Healthcare: How to implement Medical Imaging using Machine Learning | Skyl.ai |
| AI in Healthcare: Can AI help us in Diagnosing Corona Virus | Skyl.ai |
| Masterclass Kunstmatige Intelligentie | Smart Health |
| SIIM Webinar | Society for Imaging Informatics in Medicine |
| AI Infrastructure at an Organization | Society for Imaging Informatics in Medicine |
| AI Implementation Examples | Society for Imaging Informatics in Medicine |
| AI in Clinical Practice | Society for Imaging Informatics in Medicine |
| Machine Learning and Artificial Intelligence in Radiology: A "Gentle" Introduction | Society for Imaging Informatics in Medicine |
| Simplifying Machine Learning to Enable Citizen Scientists | Society for Imaging Informatics in Medicine |
| Assessment Methods and Regulatory Frameworks for Quantitative Imaging and Machine Learning Tools | Society for Imaging Informatics in Medicine |
| Driving AI Adoption in Clinical Practice | Society for Imaging Informatics in Medicine |
| Deep Learning for Medical Image Interpretation | Society for Imaging Informatics in Medicine |
| Challenging Trust: What do Competitions Need to do to Build Confidence in Imaging AI? | Society for Imaging Informatics in Medicine |
| 1: Introduction on AI, Machine Learning, and Deep Learning with focus on DL methods and Architectures | Society of Nuclear Medicine and Molecular Imaging |
| 2: Hands-on training for AI | Society of Nuclear Medicine and Molecular Imaging |
| 3: Radiomics, its link to Machine Learning and Deep Learning, and Uses in Predictive Modeling | Society of Nuclear Medicine and Molecular Imaging |
| 4: How Machine Learning Will Change Clinical Radiology and Nuclear Medicine | Society of Nuclear Medicine and Molecular Imaging |
| 5: Emerging uses of AI by Industry for Radiology and Nuclear Medicine | Society of Nuclear Medicine and Molecular Imaging |
| 6: Translating AI Approaches to Clinical Cardiovascular Imaging | Society of Nuclear Medicine and Molecular Imaging |
| COVID-19 and AI: A virtual conference | Stanford Center for Continuing Medical Education |
| Artificial Intelligence and Machine Learning in MSK Radiology | Stanford Center for Continuing Medical Education |
| Applied Machine Learning in Diagnostic Imaging | University Hospital Zürich |
| COVID-19: Medical Imaging Updates and Lessons Learned | University of Toronto |
| Artificial Intelligence: Hype, Reality and Future Implications for Diagnostic Imaging | UT Southwestern Radiology |
| CT Lung Image Analysis and Artificial Intelligence | VUB Artificial Intelligence Lab |
| The Continuing Challenge of AI & Data Protection Law amid the COVID-19 Crisis | VUB Artificial Intelligence Lab |
